# Perceptions of busyness in the emergency department: an opportunity to address a training gap through competency based education

**DOI:** 10.1007/s40037-017-0387-4

**Published:** 2017-11-23

**Authors:** Glen Bandiera, Kaif Pardhan

**Affiliations:** 1 St. Michael’s Hospital, University of Toronto, Toronto, Ontario Canada; 2 Emergency Department, Sunnybrook Health Sciences Centre, Toronto, Ontario Canada

In this issue, Chan et al. report interesting perspectives on the notion of ‘busyness’ in emergency departments (EDs) [1]. Specifically, they found that faculty and junior residents differed significantly in their conception of busyness and that faculty development around teaching managerial skills is deficient. We feel that the authors have outlined both an issue that cuts to the heart of what it means to be an emergency physician and an opportunity offered up by the imminent migration to more competency-based educational (CBE) approaches.

Management of complicated patient presentations and flow issues are core competencies of emergency physicians [2, 3]. The Canadian Association of Emergency Physicians defines emergency medicine as (emphasis ours): ‘a field … comprised of a *unique set of competencies* required for the *timely* evaluation, diagnosis, treatment and disposition of *all *patients with injury, illness and/or behavioural disorders requiring expeditious care, *24/7/365’* [4]. Modern emergency medicine curricula include such core competencies as ‘Demonstrate knowledge of and utilize specific strategies to manage emergency department crowding’ and ‘Facilitate management of unexpected surges in patient numbers and/or acuity’ (Canada) or ‘Employs task switching in an efficient and timely manner in order to manage the ED’ and ‘Participates in strategies to improve healthcare delivery and flow’ (US) [5, 6]. We posit that the major system ‘value-add’ of emergency physicians is precisely their acquisition of these specific competencies, leading to their ability to provide a breadth of high quality acute care in conditions of uncertainty at all hours and thus act as a volume ‘buffer’ for the system. However, multiple factors might limit the ability of caregivers and departmental resources to safely manage the load. EDs are among the few services in health care that truly have no ability to control incoming patient volumes. When ‘downstream’ backlogs create an inability to move patients to inpatient locations, ED crowding worsens. Paradoxically, while advances in ED care (such as bedside ultrasound, better procedural sedation, and accessible definitive testing including CT and high sensitivity troponin) mean more can be done in EDs and admissions may be avoided, these advances will likely prolong ED stays, contributing to congestion. We argue that the true benefit of the modern emergency physician’s skillset may be wasted with the ongoing congestion caused by overcrowding, and can only be optimized when supported by effective systemic flow initiatives.

The finding that junior residents identified different concepts of busyness than staff physicians should not surprise, with data showing junior residents focused on individual patient encounters and staff physicians acutely aware of the aforementioned system pressures. As they progress through training, we expect that residents will apply fundamental knowledge and skills to ever more complex situations, eventually mastering the breadth of foreseeable specialty-specific circumstances. Junior residents more often identified the stresses of individual patient encounters, including medical, socioeconomic and cultural complexities. One can envision such systems-level demands as regular interruptions, delicate negotiations around admissions, ED crowding and stewardship of scarce resources within the ED as complicating factors. These were identified in the faculty perceptions. For most individuals on a learning trajectory, the goal just beyond reach is more indelible than distant goals. Given that Chan et al.’s study involved mostly junior learners, it is realistic to think that they are rightly focused on their immediate stretch goal, managing the individual patient, rather than broader system level issues.

The juxtaposition of the junior resident paradigm with the systems challenges faced by faculty presents an opportunity for both educators and EDs to consider. The gap between junior residents and faculty identified by Chan et al. is not currently addressed within the educational construct of emergency medicine training. They have suggested multiple curricular changes and faculty development to address it. We posit that, with appropriate culture change, the roll-out of CBE models provide an excellent opportunity to do this in a concerted fashion and to create faculty that can manage, and find solutions for, system pressures. [7]. Time and human resources are required to both develop the explicit competencies, and support the shift in focus to learner-driven, frequent, targeted assessments that inform deliberate promotion decisions. Faculty member engagement will be particularly important in the face of the aforementioned increases in patient volumes, complexity and expanding scope of emergency physician roles.

One of the key principles of CBE is that learners should be allowed to focus on stage-appropriate goals until they demonstrate that they are ready to move on to more sophisticated issues. Training paradigms do outline clear progression of responsibility across levels. The International Federation of Emergency Medicine core curriculum project states, ‘trainees must have a chance to assume greater responsibilities throughout the educational program and provide more complex care and departmental administrative management as their skills grow’ [3]. The Royal College of Physicians and Surgeons of Canada training requirements for emergency medicine specify: ‘The senior resident must be regularly entrusted with the overall responsibility for the clinical management of the emergency department and of overall patient flow’ [5]. The more we understand how learners view their learning challenges in these domains and prepare faculty to respond, the more likely we are to get the expectations right and successfully make the transition to CBE.

How we pursue and encourage residents to achieve management and leadership competencies will need to shift to accommodate this transition. Ericsson argues that to achieve expertise we should move beyond time-based approaches, wherein residents spend large amounts of time in the ED and thus get significant domain-related knowledge. As a community of practice, we must develop well-established goals for more advanced competencies, provide targeted feedback and provide opportunities for repetition and refinement of specific skills [8]. In essence, we need to embrace a deliberate practice model, recognizing that these management skills will continue to develop after residents’ formal training is complete.

Similarly, not everything needs to be done in sequence, nor does all training need to occur in clinical milieus. One key design element of a true CBE model is the ability of residents to develop core competencies in their discipline regardless of where they are in training. In the updated 2015 Canadian CanMEDS Specialty training framework, there is a focus on the Leader Role for the first time [9]. This is an explicit endorsement that leadership competencies are important and should be fostered within residency education. This will allow residents to seek out experiences that will impart those skills to them outside the clinical environment and bring those skills back to enrich their emergency medicine community. Placement of certification examinations (which are heavy on individual patient management and memorized knowledge) in the middle, rather than at the end, of training, will finally enable programs to create what has been sorely lacking in residencies: a true and meaningful ‘Transition to Practice’ stage where some of these more sophisticated competencies can be taught. In emergency medicine, this shift means residents can be taught the nuances of department management and systems complexity without the burden of exam preparation and anxiety around failure. With that, maybe we can finally move on from a system in which we train residents to be great residents and instead train them to be great independent practitioners.
